# Impact of Bone Metastases and Actionable Genetic Alterations in Biliary Tract Cancer

**DOI:** 10.3390/cancers17101639

**Published:** 2025-05-12

**Authors:** Karim Hussien El-Shakankery, Joanna Kefas, Ramis Andaleeb, Paula Muehlschlegel, John Bridgewater

**Affiliations:** 1 Department of Medical Oncology, University College London Hospital, London NW1 2BU, UK; 2 University College London Cancer Institute, London WC1E 6DD, UK

**Keywords:** cholangiocarcinoma, biliary tract cancer, bone metastases, genetic alterations, precision oncology

## Abstract

**Background**: Bone metastasis (BM) prevalence is underreported in biliary tract cancers (BTC). This study aimed to assess BM prevalence in a real-world BTC population, alongside examining its relationship to prognosis and genomic alterations. **Methods**: Patients with histology-proven BTC as reviewed at a university cancer centre between January 2019 and August 2022 were assessed. Data extracted from records included BTC subtype, molecular profiling and systemic anti-cancer therapy (SACT) use. Stratification by BTC subtype and metastasis sites occurred. Median overall survival (mOS) was defined as time from relapse or metastases to death. Survival analysis was conducted using the Cox Proportional Hazard model. **Results**: Of 197 patients, 74 (37.6%) had intrahepatic and 67 (34%) had extrahepatic cholangiocarcinoma. Thirty-four patients had BM (17.3%), with 14 identified at initial diagnosis. OS was not influenced by bone (HR 1.15; *p* = 0.48) or liver metastases (HR 1.09; *p* = 0.6). Stratifying for age and gender, no significant difference in OS was observed. Actionable alterations were equally likely in patients with (52.4%) and without BM (58.5%). Age of BTC onset (<65 or ≥65) did not significantly influence prevalence of actionable alterations. Patients receiving matched, targeted SACT had a mOS of 29.9 months, compared to 13.3 months in those with actionable alterations but no SACT matching (HR 0.35; *p* < 0.005). **Conclusions**: In advanced BTC, BM do not affect OS. Across all cohorts, actionable alterations improved OS when treated with matched SACT.

## Simple Summary

We know little about how common bone metastases (cancer spreading to bones from its original site) are in biliary tract cancers (BTC), or if they are more common in some BTC types compared to others. To learn more, we reviewed the medical notes of 197 patients with BTC treated in a large UK cancer centre. We studied details of patients’ BTCs, if they had bone metastases, and how they responded to treatment. In patients with incurable BTC, we found no difference in survival for patients with bone metastases compared to those without. Patients with special cancer-related genetic alterations (called ‘actionable alterations’) were just as likely to have bone metastases than those without, and with similar survival rates. When targeting these alterations with matched anti-cancer treatments, over 50% of patients had a survival of over 29.9 months since their cancer diagnosis, which is longer than that reported in many other studies.

## 1. Introduction

Biliary tract cancers (BTC) represent just 1% of all cancers [1,2,3]. Although the term is often used interchangeably with cholangiocarcinoma, BTC also include gallbladder cancers (GBC) and ampullary cancers with pancreatobiliary differentiation [3,4]. Cholangiocarcinoma is subcategorised into intrahepatic (iCCA) and extrahepatic cholangiocarcinoma (eCCA), with eCCA further divided into peri-hilar and distal [2,3,4].

Frequently diagnosed at advanced or inoperable stages, BTC patients have poor prognoses and limited responses to systemic anti-cancer therapies (SACT) [2,3]. Even with curative treatments, over 50% of patients relapse [4]. Across all stages, the five-year overall survival (OS) remains below 20% [4]. Considering the limited efficacy observed with licensed SACT, studies assessing the efficacy of alternative, herbal and plant-based agents are also increasing in number, including randomised trials with large sample sizes [5,6].

While metastases to lymph nodes, lungs and liver are well-documented, particularly in cholangiocarcinoma, the prevalence of bone metastases (BM) remains poorly understood [7,8]. The available literature is often limited to studies on iCCA or data from the Surveillance, Epidemiology, and End Results (SEER) programme, which may lack granularity [7,8,9,10,11,12,13]. Additionally, landmark BTC clinical trials did not report metastatic disease sites in participants with advanced disease [14,15,16]. Little is known about the impact BM have on survival outcomes, or their relationship with actionable molecular aberrations [7,9,10,12,13].

Using a real-world BTC cohort, this study aimed to estimate the true prevalence of BM, evaluate survival outcomes with standard-of-care treatments, and explore the relationship between BM, prognosis and genomic alterations.

## 2. Materials and Methods

This study aimed to characterise the prevalence of BM in a real-world BTC population and compare survival outcomes with those reported in both randomised controlled trials (RCT) and observational cohorts. Additionally, we examined whether BM presence influenced OS and the frequency of actionable genomic alterations.

All patients with histologically confirmed BTC assessed or treated at University College London Hospital NHS Foundation Trust between January 2019 and August 2022 were included. BTC diagnoses were categorised as follows: iCCA, eCCA, cholangiocarcinoma not otherwise specified (NOS), ampullary cancer, and GBC. Data were collected from electronic health records and chemotherapy databases.

Data extracted included: demographics, BTC subtype, TNM staging, sites of metastases (at diagnosis and during disease course), operability status, molecular profiling, SACT use, clinical trial participation, presence of BM-related and non-BM-related hypercalcaemia, presence of skeletal-related events in those with BM, diagnosis date, relapse and survival timepoints. Metastasis sites were confirmed using cross-sectional imaging, including CT, MRI and/or FDG-PET modalities. Molecular profiling was defined as next-generation sequencing on serum or tissue samples, conducted locally or accessed via commercial or trial-based services.

‘Actionable’ molecular alterations were defined as those with matched targeted treatments with evidence of efficacy in peer-reviewed literature, accessible through the English National Health Service (NHS), RCT or pharmaceutical compassionate access programs. This study considered the following alterations as actionable: *BRCA* alterations, *FGFR* fusions or mutations, *KRAS* G12C mutation, *HER2* overexpression or mutation, *IDH1* mutation and *BRAF* V600E mutations. Clinical trial involvement data were extracted from trial records. Data was stored following University College London Hospital NHS Foundation Trust and NHS England data protection rules.

Following data anonymisation, analyses were conducted using Microsoft Excel (version 16.96.1), R (version 4.2.3), and RStudio (version 2022.12.0.353). Continuous variables were summarised using means and standard deviations. Categorical variables were summarised using medians and interquartile ranges. Outcomes were stratified by multiple variables, including age, gender, BTC subtype, metastasis sites, presence of actionable alterations, SACT received and clinical trial involvement. Curative treatment was defined as successful surgical resection, with or without adjuvant SACT.

Survival analyses were conducted for the incurable population. For inoperable and de novo metastatic patients, OS was defined as time from primary diagnosis to death of any cause. For the relapsed population, OS was defined as time from relapse to death of any cause. Survival analysis was performed using the Cox Proportional Hazard model, taking censorship into account. Survival data were displayed using Kaplan–Meier curves. Categorical variables were compared using the Chi-squared test. Statistical significance was set at *p* < 0.05.

This service evaluation was performed as an audit within University College London Hospitals NHS Foundation Trust. Only retrospective data collected as part of routine standard care was included. As per local guidelines, research ethics council approval or informed patient consent was not required.

## 3. Results

### 3.1. Patient Characteristics

**Figure 1:**
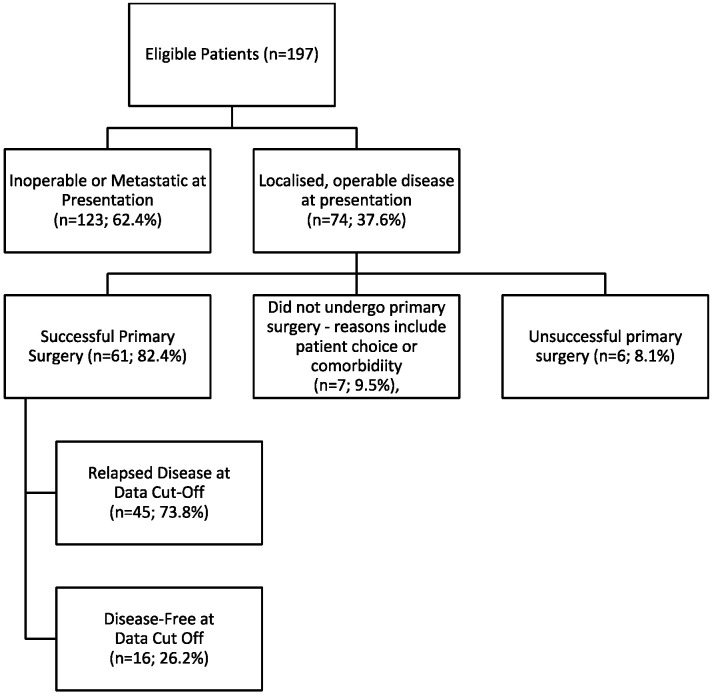
Consort Diagram Outlining Disease Status of Included Patients.

Between January 2019 and August 2022, 197 BTC patients were identified—71 patients were local to UCLH, while 126 were referrals for clinical trials or second opinions. The most common histological subtype was cholangiocarcinoma (n = 142; 72.1%). Table 1 lists population demographics and characteristics. At diagnosis, only 74 patients (37.6%) presented with resectable disease (Figure 1; Table 1), with 61 (82.4%) proceeding to surgery and 32 (52.5%) receiving adjuvant SACT (26 received capecitabine; 42.6%). Reasons for not receiving adjuvant therapy included patient choice, poor post-operative performance status, early post-operative disease progression or inoperable disease discovered intraoperatively. Overall, 168 patients were defined as incurable BTC for subsequent survival analyses, based on inoperable, metastatic or relapsed disease.

### 3.2. Cohort Survival Analysis

**Figure 2:**
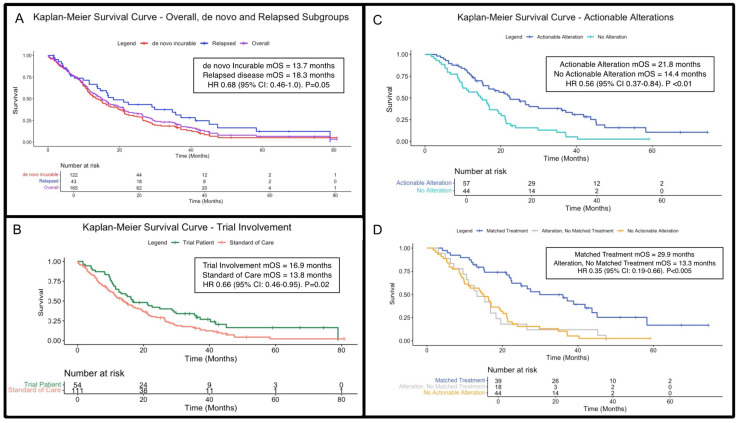
Kaplan–Meier overall survival (OS) curves for patients with incurable disease. (**A**) OS curves for the incurable cohort (purple), alongside de novo incurable (red) and relapsed disease (blue) subgroups (*p* = 0.054). (**B**) OS for clinical trial enrolment (green) vs. standard-of-care treatments (orange; *p* = 0.02). (**C**) OS stratified by presence (blue) and absence (turquoise) of actionable genetic alterations (regardless of whether patients received matched, targeted treatments; *p* < 0.01). (**D**) OS further stratified by receipt of treatment matched to actionable alterations, if applicable (*p* < 0.005). Abbreviations: BTC = biliary tract cancers; HR = hazard ratio; OS = overall survival; mOS = median overall survival.

Of the 123 patients with inoperable or metastatic disease at primary diagnosis, 122 had adequate survival data. Median OS was 13.7 months (95% CI 11.2–19.2 months; Figure 2A). Forty-three patients with relapsed disease had sufficient outcome data and a median OS of 18.3 months (95% CI 13.8–37.2 months). There was no statistical difference in OS between these groups (HR 0.68; 95% CI: 0.46–1.0; *p* = 0.05). For the overall incurable cohort (inoperable, de novo metastatic and relapsed patients), 165 patients had sufficient outcome data; median OS was 15.1 months (95% CI: 12.5–19.5 months; Figure 2A). No survival differences were observed when patients in the incurable cohort were stratified by age (<65 versus ≥65) and gender (Figure S1 and Figure S2, respectively).

The clinical trial participation rate was 30.5% overall. Patients with incurable BTC enrolled in clinical trials (n = 54 with sufficient outcome data) had a significantly longer median OS of 16.9 months (95% CI: 12.5–29.8), compared to 13.8 months (95%CI: 11.2–19.2) for those receiving standard treatment (n = 111; HR 0.66, 95%CI: 0.46–0.95, *p* = 0.02; Figure 2B). Table S1 lists included trials, including enrolment numbers.

### 3.3. Actionable Genetic Alterations

Of the 108 patients who underwent genetic profiling, all had incurable BTC and 59 (54.6%) had actionable alterations (Table 2). Stratified by age at diagnosis, a higher proportion of patients in the <65 years group had identified actionable genetic alterations, compared with those in the older group (65 or older; 61.0% vs. 52.3%), though this did not reach statistical significance (*p* = 0.49; Table 2).

Overall, 57 patients had actionable alterations and survival outcomes with a median OS of 21.8 months (95% CI: 15.3–36.0), compared to 14.4 months (95% CI: 9.83–20.1) in the 44 patients without actionable alterations and available survival data (HR 0.56; 95% CI: 0.37–0.84; *p* <0.01; Figure 2C).

Among patients with actionable alterations, 69.5% subsequently received matched targeted treatment, with a median OS of 29.9 months (95% CI: 22.5–45.1; Figure 2D) compared to 13.3 months (95% CI: 10.5–19.5) for patients with actionable alterations who did not receive targeted treatments (HR 0.35 [95% CI 0.19–0.66]; *p* < 0.005).

### 3.4. Receipt of Systemic Anti-Cancer Therapies and Associated Outcomes

**Figure 3:**
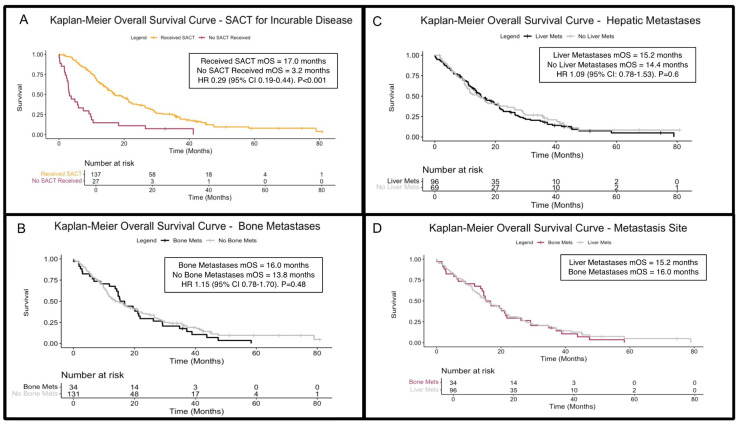
Kaplan–Meier overall survival (OS) curves for patients with incurable disease. (**A**) OS curves for patients receiving (amber) or not receiving (maroon) systemic anti-cancer therapies in the incurable (metastatic/inoperable/relapsed) setting (*p* < 0.001). (**B**) OS stratified by the presence (black) and absence (grey) of bone metastases (*p* = 0.48). (**C**) OS stratified by the presence (black) and absence (grey) of liver metastases (*p* = 0.6). (**D**) Comparison of OS in patients with liver metastases (grey) to those with bony metastases (purple), showing comparable outcomes. Abbreviations: HR = hazard ratio; mets = metastases; SACT = systemic anti-cancer therapy/therapies; mOS = median overall survival.

A total of 139 patients (82.7%) with incurable disease received SACT in metastatic and relapsed disease settings, and 137 had available outcome data with a median OS of 17.0 months (95% CI: 15.1–21.4). In comparison, those with sufficient survival data who did not receive SACT (n = 27) had a median OS of 3.2 months (95% CI: 2.8–9.2), a statistically significant difference (HR 0.29 [95% CI: 0.19–0.44]; *p* < 0.001; Figure 3A), and 83.7% (n = 103) and 82.2% (n = 37) received SACT in the metastatic/inoperable and relapsed disease settings, respectively. The reasons for not receiving palliative SACT were primarily poor performance status or patient choice. Focusing on those receiving platinum/gemcitabine combinations with sufficient outcome data, the median OS was 17.0 months (n = 126; 95% CI: 15.1–21.8) compared to 5.9 months (95% CI: 3.8–11.2) in patients not receiving platinum/gemcitabine (n = 39; HR 0.38 [95% CI 0.26–0.57]; *p* < 0.001).

### 3.5. Bone Metastases, Liver Metastases and Relation to Overall Survival

Bone metastases were identified in 34 patients (17.3%) during their disease course, with 14 cases detected at first presentation (Table 3). Patients with BM had a median OS of 16.0 months (95% CI: 14.2–21.8), while 131 patients without BM had sufficient survival outcome data and a median OS of 13.8 months (95% CI: 11.5–21.1). There was no significant survival difference between the two groups (HR 1.15; 95% CI: 0.78–1.70; *p* = 0.48; Figure 3B). When stratifying by time of BM onset, those with BM at initial presentation had a median OS of 14.8 months (95% CI 2.8–29.2); those developing BM later in their disease course had a median OS of 19 months (95% CI 14.2–37.2). The survival difference did not reach statistical significance (Figure S3; HR 2.0; 95% CI 0.97–4.15; *p* = 0.06).

To better understand the morbidity associated with BTC-induced BM, we assessed the prevalence of skeletal-related events and symptoms. Eighteen (52.9%) of the 34 patients reported bone pain and 15 (44.1%) had evidence of pathological fractures. Sixteen patients (47.1%) with BM received radiotherapy, with the intent of symptom control. Of the four patients who experienced metastatic spinal cord compression, all received radiotherapy to treat this, with one patient also undergoing neurosurgical intervention. Two other patients underwent surgery for femoral metastatic deposits. BM-associated hypercalcaemia occurred in three patients (8.8%). Alongside those receiving systematic therapies (bisphosphonates or denosumab) for hypercalcaemia, eight patients also received these for the presence BM-related symptoms or fracture prevention.

In those who underwent genetic profiling, actionable alterations were equally likely in the presence and absence of BM (52.4% vs. 58.5%; *p* = 0.95). For patients with both BM and actionable alterations (n = 11), the median OS was 19.5 months (95% CI: 15.2–not assessable). For patients with BM and no alterations (n = 10), the median OS was 14.4 months (95% CI: 6.7–not assessable). There was no significant survival difference between these groups (HR 0.95; 95% CI: 0.69–1.32; *p* = 0.8).

Of the 102 (51.8%) patients with liver metastases (LM), 61 cases (31.0% of total cohort, 59.8% of those with LM overall) were detected at diagnosis (Table 3). Of those, 96 patients had LM and sufficient outcome data, with a median OS of 15.2 months (95% CI: 13.2–21.1), compared to 14.4 months (95% CI: 10.5–23.9) without LM (n = 69). Comparing these cohorts, the difference was not significant (HR 1.09 [95% CI: 0.78–1.53]; *p* = 0.6; Figure 3C). Survival for patients with BM and LM was comparable (Figure 3D).

## 4. Discussion

### 4.1. Cohort Generalisability

In this real-world BTC cohort, subtype distribution was comparable to those in the ABC-02 (NCT00262769), ABC-06 (NCT01926236), and TOPAZ-1 trials (NCT03875235; [14,15,16]). In ABC-06 and TOPAZ-1, approximately 50% of patients had iCCA, a greater proportion than in our study. While TOPAZ-1 equally recruited from Asia, Europe, and the USA, both ABC-02 and ABC-06 were UK-based trials. The BTC subtype proportions in our cohort showed similarities to an English descriptive study of over 50,000 BTC patients identified through the National Cancer Registration Dataset, encompassing patients from diverse socioeconomic and ethnic backgrounds across all NHS England hospitals [1]. Although OS was assessed, the lack of stratification by treatment intent and metastases presence generally limits direct comparisons with this study.

Our cohort included more females than males, both in the overall and incurable populations. Although overall there was an almost equal split of patients aged <65 versus ≥65, in the incurable population more patients were aged >65. These observations are similar to those reported in other European large observational studies; survival outcomes in these subgroups are discussed further below [1,17].

Similar to another UK-based BTC study showing that higher emergency presentation rates at diagnosis correlated with a lower likelihood of curative surgery [18], most patients in this English cohort presented via emergency care pathways, which are generally associated with advanced disease stages and poorer outcomes. Although in our study, route of presentation and referral data was not collected, 62.4% of our patients had inoperable or metastatic disease at diagnosis, suggesting late presentation, likely via emergency pathways.

### 4.2. Survival in Incurable Cases—Systemic Anti-Cancer Therapy Use

In our study, only 37.6% of BTC patients presented with operable disease, consistent with previous reported estimates of 35% or lower [19,20]. Additionally, 73.8% of patients in our study relapsed after surgery, with or without adjuvant treatments [4,20,21,22]. This is higher than in the BILCAP trial, where five-year relapse rates were 66% with adjuvant capecitabine and 69% with observation. Only 52.5% of our cohort received any adjuvant SACT post-resection, with 42.6% receiving adjuvant capecitabine. The updated BILCAP survival results were not published until 2022, which may explain the less frequent use of adjuvant capecitabine in our study, potentially contributing to our observed higher relapse rates [22].

Patients receiving platinum/gemcitabine in the incurable setting in our study had a median OS of 17.5 months, exceeding survival estimates from early trials including ABC-02 [15]. Though this could be considered to reflect the increasing availability of subsequent treatment lines, it is not fully explained, as the recently published TOPAZ-1 trial also reported a lower median OS of 11.3 months in their platinum/gemcitabine control arm [14,23]. Our survival estimates also surpass reports in other observational studies, though direct comparisons are challenging, as studies include variable BTC subtypes, and some predate the use of cisplatin and gemcitabine, a standard first-line therapy [10,12,24].

Compared to large cohort studies and real-world datasets, treatment rates were higher in our tertiary centre, with 82.7% of patients with incurable disease receiving SACT (excluding adjuvant chemotherapy). In contrast, a European Network for the Study of Cholangiocarcinoma Registry study of 2334 patients found that only 26.2% of those with unresectable disease received chemotherapy, with a median OS of 10.6 months. However, this network study focused on selected centres and excluded gallbladder and ampullary cancers, so may not be representative of wider European populations [8]. SEER data from the USA estimated that 55.7% of patients with metastatic GBC received SACT [10].

An English Cancer Registry study of almost 9000 cholangiocarcinoma patients found that only 19.9% of non-operative patients received SACT [18]. Among those with initially resectable disease, the study did not differentiate between adjuvant and palliative SACT, limiting direct comparison with our study. We acknowledge that as our centre is a leading research institution, this may have influenced treatment access and outcomes.

### 4.3. Survival in Incurable Cases—Age and Gender Stratification

When stratifying for age and gender, no difference in OS was noted in our study (Figure S1 and Figure S2). Interestingly, Tataru et al.’s English, registry-based study (mentioned above) reported superior OS in both male and younger patients [1]. Two different, US-based, large studies of cholangiocarcinoma patients have also demonstrated superior OS in patients aged <50, compared to those over 50. One of these studies assessed survival in those with incurable disease, noting age to be an independent variable influencing survival [25], whereas the other study showed age to independently predict OS across all disease stages [26]. Indeed, the lack of OS difference regarding both gender and age in our study may be explained by a lower sample size, inclusion of patients with gallbladder and ampullary cancers and/or the highly selected patient cohort in this tertiary centre compared to the aforementioned studies, which are either large multi-centre studies or based on national registry data.

### 4.4. Molecular Profiling

In our study, over 50% of patients underwent genetic profiling, with 54.6% identified as having an actionable, targetable alteration. Even among patients without actionable alterations, the median OS exceeded 14 months, likely reflecting this group’s overall fitness, particularly as access to profiling is often through eligible enrolment into clinical trials. Consistent with previous findings, our data suggest that the presence of an actionable alteration alone did not confer a prognostic advantage without a matched targeted treatment [27]. However, when matched to targeted treatment, the median OS was 29.9 months (95% CI 22.5–45.1)—one of the highest reported to date [27,28].

Studies assessing differences in outcomes between younger and older patients with BTCs are increasing in number, proposing a possible relationship between age of cancer onset and underlying molecular driver mechanisms [25,26]. In keeping with results from large, US-based studies of cholangiocarcinoma patients [25,29], we show that the proportion of actionable mutations is influenced by age of diagnosis, though this did not reach significance in our cohort; small sample sizes may have influenced this. Though Pappas et al. [25] did not report the proportion of patients with actionable alterations by age group, they did show that younger patients had a higher frequency of targeted therapy use, alongside a higher frequency of *FGFR2*, *BRAF* and *ATM* alterations. When considered alongside their finding that OS was greater in patients <50 years old, this further highlights a possible association between age, actionable genetic alterations and OS. These findings are supported by a study of over 9000 iCCA patients with available tissue genomic profiling data, reporting that *FGFR2* rearrangements increase in frequency in those <45, particularly in females [29]. As just 19 patients were aged <45 in our cohort, and only 12 of these had molecular profiling, we did not explore this age cut-off further. Our findings, showing that OS was not influenced by age alone, may be explained by the lack of a significant difference between the proportion of actionable alterations between younger and older age groups. Indeed, this relationship should be assessed further in future studies.

Even in our tertiary centre with access to a broad clinical trial portfolio, 45% of patients did not undergo molecular profiling. Following UK health technology assessment approval of pemigatinib and ivosidenib in late 2021 and 2024, respectively, standard-of-care genetic testing for *FGFR2* and *IDH*-1 is now provided on the NHS [30,31,32,33]. Our study largely predated these events. Until recently, whole genome sequencing was only available through clinical trials or private funding. Since 2023, the NHS England National Genomic Testing Directory permits molecular profiling on NHS BTC patients. However, to qualify for testing, patients must have exhausted all standard-of-care treatments, therefore assuming them to be metastatic [34]. No equivalent testing is currently available for NHS Scotland patients, and so any testing outside of trial profiling must be personally and privately funded [35]. Furthermore, access to matched targeted agents in patients with atypical identifiable alterations remains a challenge UK-wide and is generally not funded by the NHS. No agreements for molecular profiling are in place for patients with resectable or curable disease, mainly due to the limited availability of licensed targeted treatments in this setting. These issues are also compounded by known limitations relating to the availability of sufficient tissue for testing in eligible populations. Indeed, when these factors are considered in the context of significantly prolonged survival in patients receiving matched targeted treatments, as shown in this study, limited access to molecular profiling within public healthcare systems results in implications for patients, primarily relating to equity of access to both molecular testing and subsequent targeted treatments in BTC cohorts.

### 4.5. Bone Metastases

Our study estimated that 17.3% of patients develop BM during their disease course, with 7.1% presenting with BM at primary diagnosis. BM incidence in BTC is sparsely reported in the existing literature, as key RCTs (such as ABC-02, ABC-06 and TOPAZ-1) did not specify sites of metastatic burden [14,15,16]. However, several observational studies have reported BM prevalence in specific BTC subgroups (Table 4; [7,8,9,10,11,12,13]). A Brazilian study including all BTC reported a similar BM prevalence of 19.2%, though it only included patients receiving first-line chemotherapy, thereby excluding supportively managed patients [12]. Across other studies, estimations of BM prevalence range from 6.2% to 29.7% depending on BTC subtype, disease stage at diagnosis and sample size. In our dataset, cholangiocarcinoma (especially iCCA) had a higher BM prevalence than GBC (Table 3), though small subgroup sizes limit definitive conclusions. Direct comparisons with other studies are also challenging due to study heterogeneity (Table 4).

Large population studies and meta-analyses have evaluated the impact of metastasis site on cancer outcomes. In breast, renal and prostate cancers, BM are associated with a worse survival than lymph node metastases, but a better survival than visceral (including liver) and central nervous system metastases [36,37,38]. In BTC, most observational studies (summarised in Table 4) suggest BM correlate with poorer prognoses across all subtypes, though methodologies vary, particularly in distinguishing isolated versus non-isolated BM [7,10,11,12,13]. In contrast, our study found BM did not significantly impact survival compared to other metastatic sites, including LM. However, even though BM presence did not influence survival in our study, we show evidence of significant morbidity and symptom burden associated with their presence, with over 50% reporting pain associated with BM and almost 50% receiving radiotherapy to bone for symptoms.

To our knowledge, this study is the first to report that patients with BM were equally likely to have actionable alterations than those without. Patients with concurrent BM and actionable genomic alterations had comparable survival to those with BM alone. However, small sample sizes in all subgroups may have affected statistical significance and survival estimates.

### 4.6. Study Strengths and Limitations

Our study is the first to examine the relationship between BM and actionable alterations, offering novel insights for future research. Despite its contributions, our study has limitations. Reliance on electronic health records restricted data availability, including no assessable time points from BM confirmation to death for survival analysis. Potential clinical coding errors may have affected patient identification, particularly in cases where patients died soon after diagnosis, leading to missing or inaccurate coding. Subgroup sample sizes were small in certain cases, influencing statistical significance and result interpretation. Finally, as this study was conducted in a tertiary cancer centre with an active clinical trial portfolio, our cohort may not be fully representative of real-world practice.

## 5. Conclusions

In patients with BTC, actionable alterations were equally likely with or without BM, and the presence of BM did not affect OS in our cohort. While actionable alterations facilitated access to targeted treatment and better survival, this benefit was not unique to patients with BM. Larger collaborative studies with standardised methodologies are needed to determine BM prevalence rates more accurately.

## Figures and Tables

**Table 1:** Real-World Study Biliary Tract Cancer Population Demographics.

Category		Result
**Age (Years)**	Mean (Standard Deviation)	63.1 (49.6–76.6)
	Range	25–90
**Gender**	Male	83 (42.1%)
	Female	114 (57.9%)
Biliary Tract Cancer Subtype *	Intrahepatic Cholangiocarcinoma	74 (37.6%)
	Extrahepatic Cholangiocarcinoma	67 (34.0%)
	Cholangiocarcinoma Not Otherwise Specified	1 (0.1%)
	Gallbladder	46 (23.4%)
	Ampulla	9 (4.6%)
	Total	197 (100%)
**Status at Primary Diagnosis**	Localised/Operable	74 (37.6%)
	Inoperable or Metastatic	123 (62.4%)
	Total	197 (100%)
**Curative Primary Resection/Surgery**	Successful	61 (31.0%)
	Unsuccessful (unexpected dissemination or inoperable disease discovered intraoperatively)	6 (3.0%)
	Feasible But Not Attempted	7 (3.6%)
	Unfeasible	123 (62.4%)
	Total	197 (100%)
**Relapse Post-Curative Treatment**	Yes	45 (22.8%)
	No	16 (8.2%)
	Not Applicable	136 (69.0%)
	Total	197 (100%)
**Bone Metastases at Any Time** **(All Stages at Primary Diagnosis)**	Yes	34 (17.3%)
	No	163 (82.7%)
	Total	197 (100%)
**Bone Metastases (Inoperable, Relapsed and de novo Metastatic Cases only)**	Yes	34 (20.2%)
	No	134 (79.8%)
	Total	168 (100%)
**Bone Metastasis-Associated Hypercalcaemia**	Yes	2 (5.9%)
	No	32 (94.1%)
	Total	34 (100%)
**Liver Metastases at Any Time** **(All Stages at Primary Diagnosis)**	Yes	102 (51.8%)
	No	95 (48.2%)
	Total	197 (100%)
**Liver Metastases (Inoperable, Relapsed and de novo Metastatic cases only)**	Yes	102 (61.0%)
	No	66 (39.0%)
	Total	168 (100%)
**Received Adjuvant Capecitabine**	Yes	26 (42.6%)
	No	35 (57.4%)
	Total	61 (100%)
**Received Adjuvant Systemic Anti-Cancer Therapy**	Yes	32 (52.5%)
	No	29 (47.5%)
	Total	61 (100%)
**Received Platinum/Gemcitabine in Incurable Setting** **(Inoperable, Relapsed and de novo Metastatic Cases Only)**	Yes–Cisplatin	122 (72.6%)
	Yes–Carboplatin	6 (3.6)
	No	40 (23.8%)
	Total	168 (100%)
**Received any Systemic Anti-Cancer Therapies in Incurable Setting (Inoperable, Relapsed and de novo Metastatic Cases Only)**	Yes	139 (82.7%)
	No	29 (17.3%)
	Total	168 (100%)
**Trial Involvement**	Yes	60 (30.5%)
	No	137 (69.5%)
	Total	197 (100%)

* Not = 100% due to rounding.

**Table 2:** Real-World Biliary Tract Cancer Population Genetic Profiling Assessment and Results.

		Actionable Alterations Identified				
		No	Yes	Result Not Known	N/A	Sum
**Genetic Profiling Undertaken**	No	0	0	0	89	89
	Yes	44	59	5	0	108
	Sum	44	59	5	89	197
**Age at Diagnosis**	<65	23	36	3	37	99
	≥65	21	23	2	52	98
	Sum	44	59	5	89	197

**Table 3:** Bone and Liver Metastases in the Biliary Tract Cancer Real-World Cohort. Breakdown by Malignant Subtype and Actionable Alteration Status.

		Bone Metastases (At Any Time)		Liver Metastases (At Any Time)		Sum
		No	Yes	No	Yes	
**Primary Site of Malignancy**	Intrahepatic Cholangiocarcinoma	56 (75.7%)	18 (24.3%)	24 (32.4%)	50 (67.6%)	74
	Extrahepatic Cholangiocarcinoma	57 (85.1%)	10 (14.9%)	40 (59.7%)	27 (40.3%)	67
	Cholangiocarcinoma Not Otherwise Specified	1 (100%)	0 (0%)	1 (100%)	0 (0%)	1
	Gallbladder	40 (87.0%)	6 (13.0%)	25 (54.3%)	21 (45.7%)	46
	Ampulla	9 (100%)	0 (0%)	5 (55.6%)	4 (44.4%)	9
**Actionable Alteration**	Yes	48 (81.4%)	11 (18.6%)	15 (25.4%)	44 (74.6%)	59
	No	34 (77.3%)	10 (22.7%)	20 (45.5%)	24 (54.5%)	44
	Not Known/Not Assessed	81 (86.2%)	13 (13.8%)	60 (63.8%)	34 (36.2%)	94
	**Sum**	163	34	95	102	197

**Table 4:** Existing Published Studies Assessing the Role of Bone Metastases in Biliary Tract Cancers.

Author	Year and Country of Publication	Population	Sample Size	Estimated Bone Metastasis Prevalence	Survival Analysis
**Felisimino** **et al.** [12]	2022 Brazil	Only inoperable, metastatic or relapsed biliary tract cancer patients who received chemotherapy.	104	19.2% (n = 20)	Overall survival (defined as date from cycle one day one of chemotherapy to death): 11.4 months (95% CI: 9.0–13.7) across whole cohort. BM independently associated with inferior survival in multivariate Cox regression: HR 3.53 (95% CI: 1.18–10.5), *p* = 0.023.
**Santini et al.** [9]	2018 Italy	Biliary tract cancers of all types and stages. All cases had BM. Multi-Centre Cohort.	137	Not Reported	Median duration from BM diagnosis to death: - Overall: 6 months (95% CI: 4.9–7.1), - Receiving bisphosphonates: 8 months (95% CI: 6.26–9.74), - Not receiving bisphosphonates: 4 months (95% CI: 3.00–4.99; *p* = 0.001).
**Garajova** **et al.** [7]	2023 Italy	Intrahepatic cholangiocarcinoma. All inoperable or metastatic. Multi-Centre Cohort	186	10.9% (n = 20)	Median Overall Survival: - Overall: 9 months (95% CI 7.4–10.6 months). - Those with BM (isolated and non-isolated combined): 4 months (95% CI 1.0–8.3 months) - Those with isolated LM: 11 months (95% CI 8.3–13.6 months) Multivariate Cox regression: Presence of BM associated with poorer outcomes (HR 2.29).
**Izquierdo-Sanchez** **et al**. [8]	2022 Spain	Cholangiocarcinoma All Stages. From the ENSCCA Registry (multicentre).	2234	Intrahepatic CCA: 13.8% (n = 34) Perihilar CCA: 6.2% (n = 8) Distal CCA: 6.3% (n = 5)	Not Analysed
**Yan et al.** [13]	2019 China	Intrahepatic cholangiocarcinoma. Stage IV cases only (Any T, Any N, M1). Using SEER Data.	981	29.7% (n = 291)	Median Overall Survival: - Overall: 6 months (95% CI: 5.43–6.56) - With BM: 4 months (95% CI: 2.95–5.05) - With LM: 6 months (95% CI: 4.95–7.05) Comparing overall survival in patients with BM and LM: *p* = 0.003
**Cheng et al.** [11]	2019 China	Intrahepatic cholangiocarcinoma. Locally advanced or metastatic. (Any T, N ≥ 1 and/or M1). Using SEER Data.	1567	14.5% (n = 227)	Comparing overall survival in patients with isolated BM and isolated LM: *p* = 0.025 Median overall survival estimates not provided.
**Gera et al.** [10]	2023 USA	Gallbladder cancer (86% adenocarcinoma; other histologies also included). Stage IV cases only (Any T, Any N, M1). Using SEER Data	2724	7.4% (n = 202) at primary diagnosis	Median Overall Survival in Ages 18–74: - Overall: 4 months (IQR 1–9) - Without BM: 6 months - With BM: 3 months Median Overall Survival in Ages ≥75: - Overall: 3 months - Without BM: 3 months - With BM: 3 months Multivariate Cox analysis: BM increases mortality risk (HR 1.50; 95% CI 1.29–1.75; *p* < 0.001).
**Pappas et al.** [25]	2023 USA	Cholangiocarnoma All Stages. Multi-Centre Cohort	263	13.3% (n = 35) at primary diagnosis	Not Analysed

Table 4 abbreviations: BM—bone metastases; CCA—cholangiocarcinoma; CI—confidence interval; ENSCCA—European Network for the Study of Cholangiocarcinoma; HR—hazard ratio; LM—liver metastases; SEER—Surveillance, Epidemiology, and End Results; USA—United States of America.

## Data Availability

Data supporting reported results can be accessed, in accordance with NHS England and University College London Hospital NHS Foundation Trust data protection rules, through direct application to the corresponding author.

## Supplementary Materials

The following supporting information can be downloaded at: https://www.mdpi.com/article/10.3390/cancers17101639/s1, Figure S1: Kaplan-Meier Overall Survival Curves for Patients with Incurable Disease, Stratified by Age; Figure S2: Kaplan-Meier Overall Survival Curves for Patients with Incurable Disease, Stratified by Gender; Figure S3: Kaplan-Meier Overall Survival Curves for Patients with Incurable Disease and Bone Metastases, Stratified by Onset of Bone Disease; Table S1: List of Enrolled Clinical Trials and Number of Enrolled Patients.
