# Supplementary material for: Impact of Bone Metastases and Actionable Genetic Alterations in Biliary Tract Cancer
Source: Cancers (Basel). 2025 May 12;17(10):1639. doi: 10.3390/cancers17101639 (PMC12110439; doi:10.3390/cancers17101639)
Supplement: Supplementary file 1 [file cancers-17-01639-s001.zip › cancers-3516072-supplementary.pdf]

MANUSCRIPT TITLE: Impact of Bone Metastases and Actionable Genetic Alterations in Biliary Tract Cancer

AUTHOR LIST: K.H. El-Shakankery, J. Kefas, R. Andaleeb, P. Muehlschlegel, J. Bridgewater.

SUPPLEMENTARY DATA

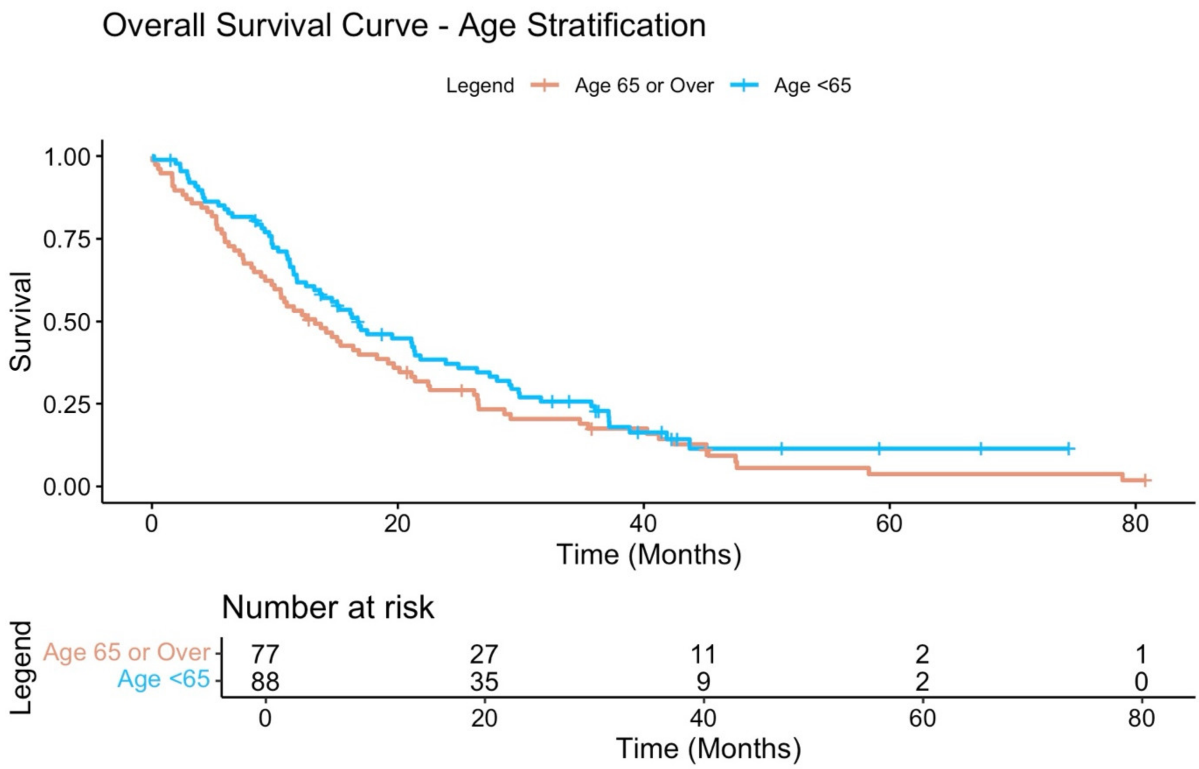

Supplementary Figure S1: Kaplan-Meier Overall Survival Curves for Patients with Incurable Disease, Stratified by Age. In total, 165 patients had available data for age and sufficient survival outcomes. Overall survival curves are shown for the Age 65 and over (orange) and the less than 65 (orange) subgroups. No significant difference between groups was observed (Hazard Ratio 1.00; 95% Confidence Interval 0.99-1.02; P=0.3).

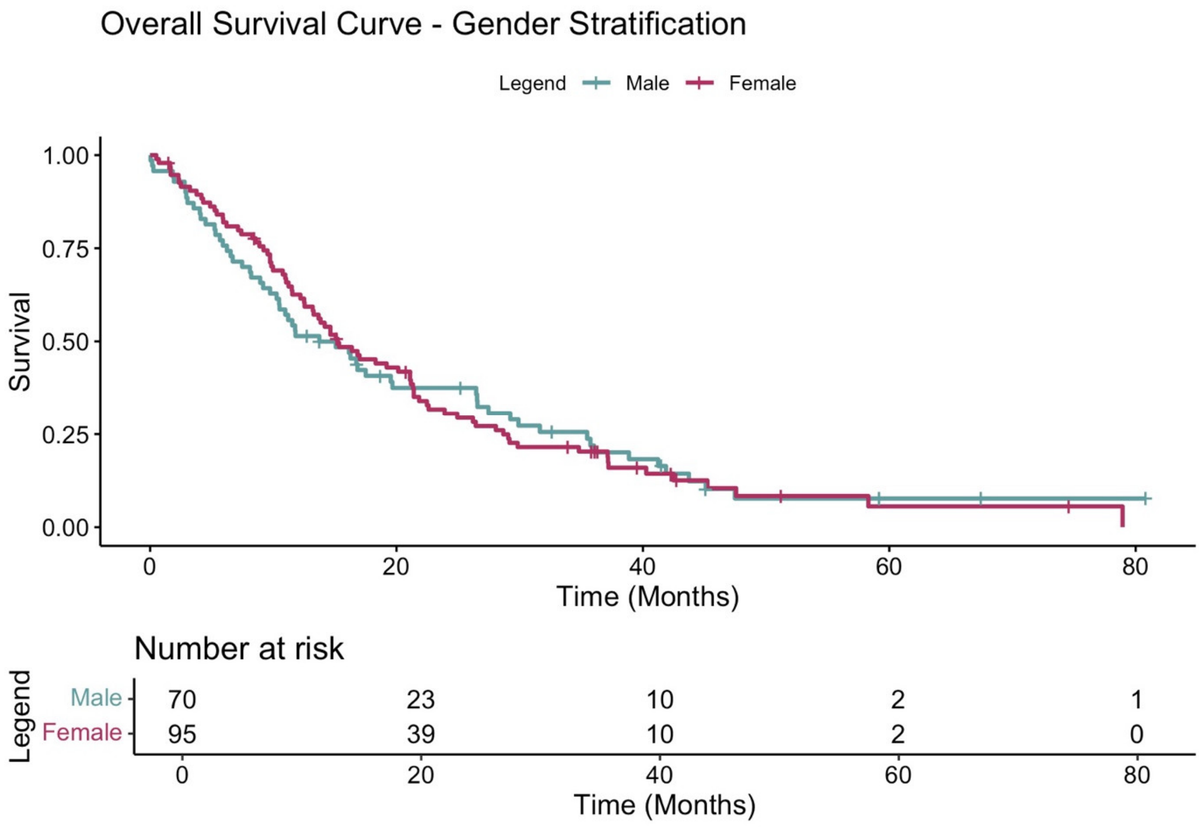

Supplementary Figure S2: Kaplan-Meier Overall Survival Curves for Patients with Incurable Disease, Stratified by Gender. In total, 165 patients had available data for gender and sufficient survival outcomes. Overall survival curves for the male (turquoise) and female (maroon) subgroups are shown. No significant difference between groups was observed (Hazard Ratio 1.0; 95% Confidence Interval 0.71-1.39; P=0.98).

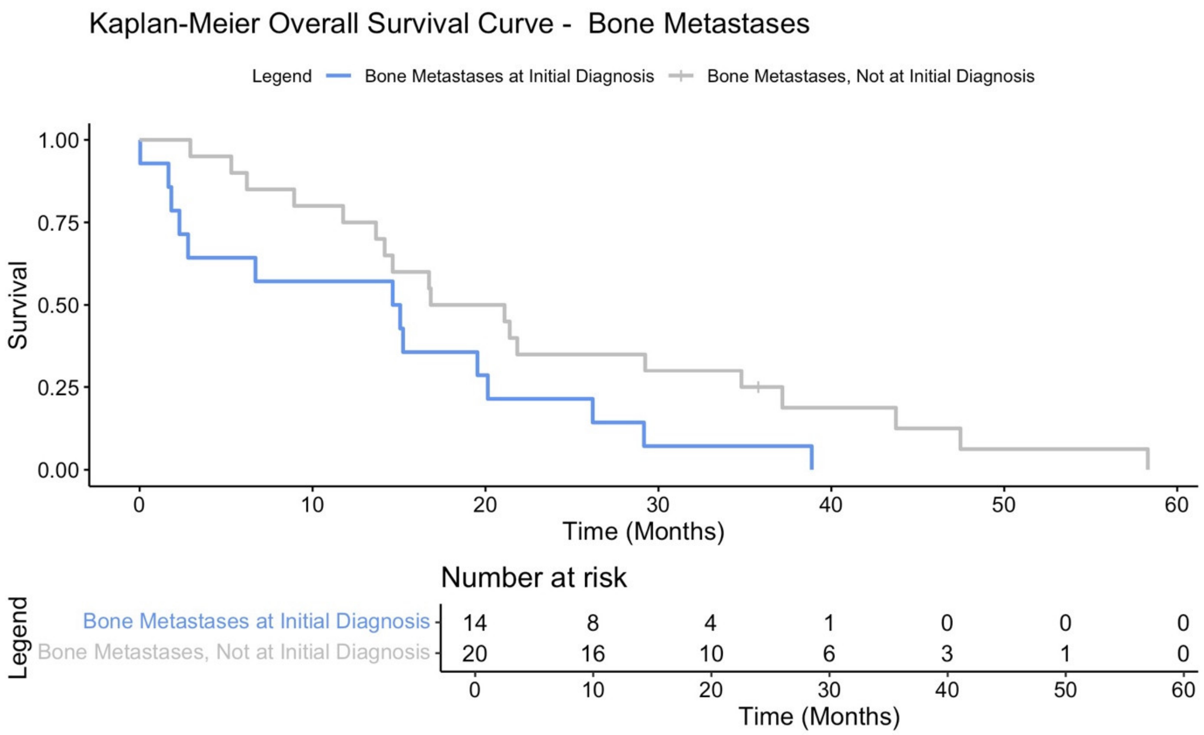

Supplementary Figure S3: Kaplan-Meier Overall Survival Curves for Patients with Incurable Disease and Bone Metastases, Stratified by Onset of Bone Disease. In total, 34 patients had available data for the presence of bone metastases and sufficient survival outcomes. Overall survival curves for the presence of bone metastases at initial diagnosis (blue), and presence at any time (grey), are displayed. No statistically significant difference between the groups was observed (Hazard Ratio 2.0; 95% Confidence Interval 0.97-4.15; P=0.06).

Trial Name	NCT	Number Recruited
ABC-07	ISRCTN10639376	12
ABC-09	NCT03260712	14
ACTICCA-1	NCT02170090	7
Agios-120 (AG120) trial	NCT02989857	2
Arginase 203 trial (Incyte)	NCT03314935	4
BAS-301	NCT04604132	1
BILCAP	NCT00363584	1
FIGHT-302 (Incyte)	NCT03656536	3
M7824 (bintrafusp alfa)	NCT03833661	1
PORCUPINE2	NCT04907851	4
RAGNAR (erdafitinib)	NCT04083976	1
ReFocus (RLY-4008-101)	NCT04526106	2
FOENIX-CCA2 (TAS-120; futibatinib)	NCT02052778	8
TOPAZ-1	NCT03875235	1
HERIZON-BTC-01 (Zymeworks)	NCT04466891	2
INCAGN01949	NCT02923349	1

Supplementary Table S1: List of Enrolled Clinical Trials and Number of Enrolled Patients. In total, 60 (30.5%) patients were enrolled on clinical trials at our tertiary cancer centre; 56 had incurable disease. Table S1 lists the names and details of all trials in which patients in this study were recruited to, alongside the number of patients recruited to each trial. Of note, some patients were recruited to more than one trial during their disease course, hence the total number of patients in Table S1 equates to over 60.
